# Metabolomic analysis of *Streptococcus pneumoniae*: uncovering key metabolic pathways

**DOI:** 10.3389/fmicb.2025.1707940

**Published:** 2025-12-04

**Authors:** Haiyan Jiang, Changliang Zhao, Lu Feng, YuJun Gao, Zhihui Mi, Haiying He

**Affiliations:** 1 Department of Paediatric, The Third Worker's Hospital of BaoGang Group, Baotou, Inner Mongolia, China; 2 Inner Mongolia Di An Feng Xin Medical Technology Co., Ltd., Huhhot, Inner Mongolia, China

**Keywords:** *Streptococcus pneumoniae*, metabolomics, galactose metabolism, hypoxia-inducible factor-1 (HIF-1) signaling pathway, immune response

## Abstract

**Background:**

Pneumococcal infection, caused by *Streptococcus pneumoniae*, is a prevalent cause of community-acquired pneumonia and a major pathogen responsible for illnesses such as meningitis, sepsis, and pharyngitis. The complex etiology of *S. pneumoniae* infection poses significant challenges in elucidating the molecular mechanisms underlying its pathogenesis.

**Methods:**

In this study, twenty serum samples from individuals infected with *S. pneumoniae* and fifteen serum samples from normal controls were analyzed using liquid chromatography/mass spectrometry (LC–MS) to identify metabolites. Multivariate statistical analyses, including principal component analysis (PCA) and orthogonal partial least squares discriminant analysis (OPLS-DA), were used to identify potential metabolites. Kyoto Encyclopedia of Genes and Genomes (KEGG) pathway enrichment analysis was employed to map the metabolic pathways associated with these metabolites.

**Results:**

Through comparative analysis of the metabolic profiles of infected individuals and normal controls, we identified 418 metabolites that significantly contributed to the differentiation of group samples. The identified metabolites were categorized into various groups, such as amino acids, fatty acids, and phosphatidylcholine, and were enriched in pathways including galactose metabolism, the hypoxia-inducible factor-1 (HIF-1) signaling pathway, the citrate cycle, the pentose phosphate pathway, and glycolysis/gluconeogenesis. *S. pneumoniae* infection induced significant variations in the serum metabolome, with activation of metabolic pathways implicated in the immune response.

**Conclusion:**

Our study provides a comprehensive and real-time analysis of the metabolic network, elucidating the complex processes that occur following pathogen invasion in the human body. The identification of metabolic biomarkers and enriched pathways lays a foundational framework for research and offers visualizable targets for the diagnosis and treatment of pneumococcal infections.

## Introduction

Community-acquired pneumonia (CAP) is characterized by infectious inflammation of the lung parenchyma, including alveolitis and interstitial pneumonia, and is contracted outside hospital settings. CAP is commonly observed in elderly individuals, children, and individuals with compromised immune systems (Meyer Sauteur, 2024). CAP is one of the leading causes of hospitalization among children in developed countries and the primary cause of mortality among children in developing countries (Jain et al., 2015; O'Brien et al., 2019; Zar et al., 2016). *Streptococcus pneumoniae* is the pathogen predominantly responsible for CAP, contributing to approximately 40 to 60% of cases and resulting in significant morbidity and mortality among children (Cui et al., 2024). Challenges persist in the management and treatment of *S. pneumoniae* infections. In the context of hospitalized patients with CAP, meticulously monitoring the response to antibiotic therapy to improve the treatment effect and optimize treatment strategies is crucial (Pletz et al., 2022). Furthermore, there is an urgent need for explicit guidelines to inform decisions regarding discontinuing antibiotic therapy early, with the aim of reducing the risk of antimicrobial resistance. The primary CAP treatment efficacy indicators include clinical symptoms, imaging findings, and levels of immune markers such as C-reactive protein (CRP) and procalcitonin (PCT) (Yadav et al., 2024). Dynamic changes in PCT levels can indicate the severity of infection and the efficacy of treatment (Cleland and Eranki, 2025). Nonetheless, a comprehensive explanation of the prognostic factors of bacterial infections remains necessary, and this requires elucidating the mechanisms underlying disease onset and progression.

Metabolomics, a crucial area in the field of systems biology, has been extensively applied in clinical contexts, particularly in disease diagnosis and treatment (Chen et al., 2024). Recent integrative metabolomic and network-pharmacologic analyses have further demonstrated how systemic metabolic remodeling accompanies infectious and traumatic inflammatory states (Li et al., 2024).

This discipline involves the study of the complete set of low-molecular-weight metabolites present within cells or organisms. Metabolites are an objective reflection of the metabolic state under specific conditions (Wang et al., 2023). In metabolomics, group indicators are analyzed and high-throughput detection and data processing techniques are utilized with the ultimate goal of achieving comprehensive information modeling and system integration (Perez de Souza and Fernie, 2024). Systems biology now provides a robust framework for integrating metabolic signatures into CAP diagnosis and therapy. For example, Chouchane et al. explored the relationships between lipid metabolism and the severity, systemic immunity, and mortality of sepsis through lipidomics, thereby providing a more systemic visualization of the progression of this illness (Chouchane et al., 2024). Additionally, some researchers have investigated metabolic differences across various stages of CAP from a longitudinal perspective, through which effective therapeutic targets can be identified while monitoring disease progression from onset to recovery (den Hartog et al., 2024). The emergence of drug resistance and the rapid evolution of pathogens in CAP present significant challenges for diagnosis, necessitating ongoing research to address existing gaps in this field. Furthermore, integrating multi-compartment metabolomics, as demonstrated in gut-brain axis models, improves the interpretation of systemic metabolic alterations (Cheng et al., 2022).

The aim of this study was to comprehensively characterize the metabolite profile of CAP patients with confirmed *S. pneumoniae* infection compared with that of noninfected individuals through metabolite profiling. Additionally, we conducted an analysis of relevant clinical indicators to evaluate the overall metabolic status of the body following CAP infection, with the aim of providing a refined diagnostic basis for the disease.

## Materials and methods

### Ethics statement

The study protocol received approval from the Ethics Committee of The Third Worker’s Hospital of BaoGang Group (approval number: BGSYY-KYLL-2024001). Written informed consent was obtained from all participants, and all subjects were anonymized to prevent identification from clinical data.

### Study subjects

The study included a total of twenty patients admitted to the pediatric department of the Third Worker’s Hospital of BaoGang Group between April 2024 and June 2024. Patients were diagnosed with *S. pneumoniae* infection in accordance with the guidelines delineated in the “Expert Consensus on Laboratory Diagnostics and Clinical Practices for *S. pneumoniae* Infection in Children in China (2020)” (China National Clinical Research Center for Respiratory Diseases et al., 2020) and the “Guidelines for the Management of Community-Acquired Pneumonia in Children (2024)” (Subspecialty Group of Respiratory et al., 2024). Nucleic acids were extracted from throat swab samples utilizing Nucleic Acid Extractor (A32mini, HealthGeneTech Co., Ltd., Ningbo) and Virus Nucleic Acid Extraction Reagent (Nucleic Acid Extraction or Purification Kit, HealthGeneTech Co., Ltd.). Primers specific to the highly conserved sequences of respiratory pathogens were identified through quantitative polymerase chain reactions (qPCR) coupled with tandem capillary electrophoresis (Sanger Sequencer T400, HealthGeneTech Co., Ltd., Ningbo). Patients presenting with mixed infections involving additional pathogens were excluded from the study. Healthy individuals without infections were recruited as normal controls.

### Sample collection and preparation

Venous blood samples (5 mL) were collected from infected individuals during their initial hospital admission for examination, allowed to clot at room temperature for 20 min, and subsequently centrifuged to remove the clot, with all procedures conducted in the morning. The resulting samples were divided into aliquots and stored at −80 °C for future use. Prior to analysis, the frozen samples were thawed gradually in an ice bath and agitated for 10 s to ensure homogeneity. For sample preparation, 50 μL of the sample was transferred into a centrifuge tube and combined with 300 μL of a methanol-acetonitrile extraction solvent (v:v = 4:1). The mixture was vortexed for 3 min to ensure thorough mixing. The tube was subsequently centrifuged at 12,000 rpm for 10 min at 4 °C. Following centrifugation, 200 μL of the supernatant was carefully extracted and stored at −20 °C for 30 min to facilitate the formation of any precipitates. After this cooling period, the supernatant was centrifuged again at 12,000 rpm for an additional 3 min at 4 °C, resulting in a clear supernatant ready for instrumental analysis.

### High-performance liquid chromatography/mass spectrometry conditions

To ensure complete separation, the sample was eluted using a Waters ACQUITY Premier HSS T3 column (1.8 μm, 2.1 mm × 100 mm) in both positive and negative modes. The analytical conditions were established as follows: the column temperature was maintained at 40 °C, with a flow rate of 0.4 mL/min and an injection volume of 4 μL. The parameters for the negative ion mode mirrored those of the positive ion mode. A solvent system comprising 0.1% formic acid in water (solvent A) and 0.1% formic acid in acetonitrile (solvent B) was employed to maximize the separation of metabolites in the samples. The optimized elution gradient was as follows: an initial increase from 5 to 60% of solvent B over 0–3 min, then to 99% within 1 min, which was held for 1.5 min. Subsequently, mobile phase B was returned to 5% within 0.1 min and maintained for 2.4 min. Data acquisition alternated between full-scan mass spectrometry (MS) and data-dependent MS2 scans utilizing dynamic exclusion. MS analyses were conducted using electrospray ionization in both positive and negative ion modes, with full-scan analysis over a mass–charge (m/z) range of 75–1,000 at a resolution of 35,000. The MS parameter settings were as follows: the ion spray voltage was set to 3.5 kV for positive mode and 3.2 kV for negative mode, the sheath gas flow rate was 30 Arb, the auxiliary gas flow rate was 5 Arb, the ion transfer tube temperature was maintained at 320 °C, the vaporizer temperature was set at 300 °C, and the collision energy ranged from 30 to 50 V.

### Data pre-treatment and statistical analysis

The initial LC–MS data were converted to mzML format utilizing Proteo Wizard software. The mzML files were subsequently processed using the XCMS program, an R-based platform specifically designed for the processing and visualization of LC–MS data. This processing encompassed peak extraction, peak alignment, and retention time (RT) correction. The MS peak list was aligned with mass tolerance and RT tolerance parameters set at 0.25 Da and 30 s, respectively, to minimize RT shifts and eliminate extraneous signals. Parameter optimization algorithms based on chromatographic peak shape can enhance reproducibility in LC–MS metabolomics (Sheng et al., 2024). The algorithm and parameters for peak detection included a minimum peak width of 5 s, a maximum peak width of 20 s, a deviation of 5 ppm, and a signal-to-noise threshold of 4. Peaks with a detection rate less than 50% within each sample group were excluded, and the resulting dataset was subjected to further analysis using various statistical modules available in XCMS. Principal component analysis (PCA) was conducted using an R package (). PCA is a widely employed technique for data dimensionality reduction, which not only reduces the number of features but also enables data visualization, thereby streamlining model computations. Orthogonal partial least squares discriminant analysis (OPLS-DA) was utilized to identify metabolites distinguishing samples across different groups by screening criteria based on variable importance in projection (VIP) values and *p* values. Metabolites with VIP values greater than 1 are considered to significantly contribute to group differences, whereas the *p* value is primarily used to assess the statistical significance of differences in means between two datasets.

### Metabolite identification

Metabolite identification was achieved through a comprehensive search of both the proprietary database of the laboratory and publicly accessible databases, including the Human Metabolome Database (HMDB)^1^, artificial intelligence (AI)-database-local libraries based on machine learning models (Allen et al., 2014) and Metabolite identification and Dysregulated Network Analysis (metDNA).

### Kyoto Encyclopedia of Genes and Genomes annotation and enrichment analysis

The identified metabolites were annotated using the Kyoto Encyclopedia of Genes and Genomes (KEGG) compound database, and subsequently, these annotated metabolites were mapped onto the KEGG pathway database.

### Statistical analysis

The statistical analysis was performed via the R Studio program (version 4.2.0), and Student’s *t* test was used to determine differences between groups. A significance level of *p* ≤ 0.05 was set to assess intergroup variations.

## Results

### Clinical information of the enrolled participants

Detailed clinical information of the enrolled participants is presented in Supplementary Table 1, including age and various infection indices, such as the white blood cell (WBC) count, platelet (PLT) count, neutrophil-to-lymphocyte ratio (NLR), PCT level, aspartate aminotransferase (AST) level, alanine aminotransferase (ALT) level, CRP level, and neutrophil percentage (NE%). The mean values for these parameters were as follows: an age of 5.26 ± 3.07 years, a WBC count of (9.85 ± 4.78) × 109/L, a PLT count of (279.48 ± 80.23) × 109/L, a NLR of 1.57 ± 1.24, an AST level of 33.32 ± 12.55 U/L, an ALT level of 14.72 ± 5.56 U/L, a CRP level of 6.01 ± 5.56 mg/L, a PCT level of 0.09 ± 0.11 ng/mL, and a NE% of 49.85 ± 18.97. In the normal control group, the mean values were as follows: an age of 5.15 ± 2.29 years, a WBC count of (7.90 ± 1.54) × 109/L, a PLT count of (230.50 ± 34.69) × 109/L, a NLR of 1.81 ± 0.94, an AST level of 26.50 ± 6.19 U/L, an ALT level of 13.40 ± 4.53 U/L, a CRP level of 3.23 ± 2.34 mg/L, a PCT level of 0.02 ± 0.02 ng/mL, and a NE% of 43.78 ± 11.65. Statistically significant differences were observed in the PLT count, AST level, CRP level, and PCT level. Compared with those in the normal control group, the PLT count and AST, CRP, and PCT levels in the *S. pneumoniae* group were elevated.

### Serum metabolomics and the consistency of analytical reproducibility

**Figure 1:**
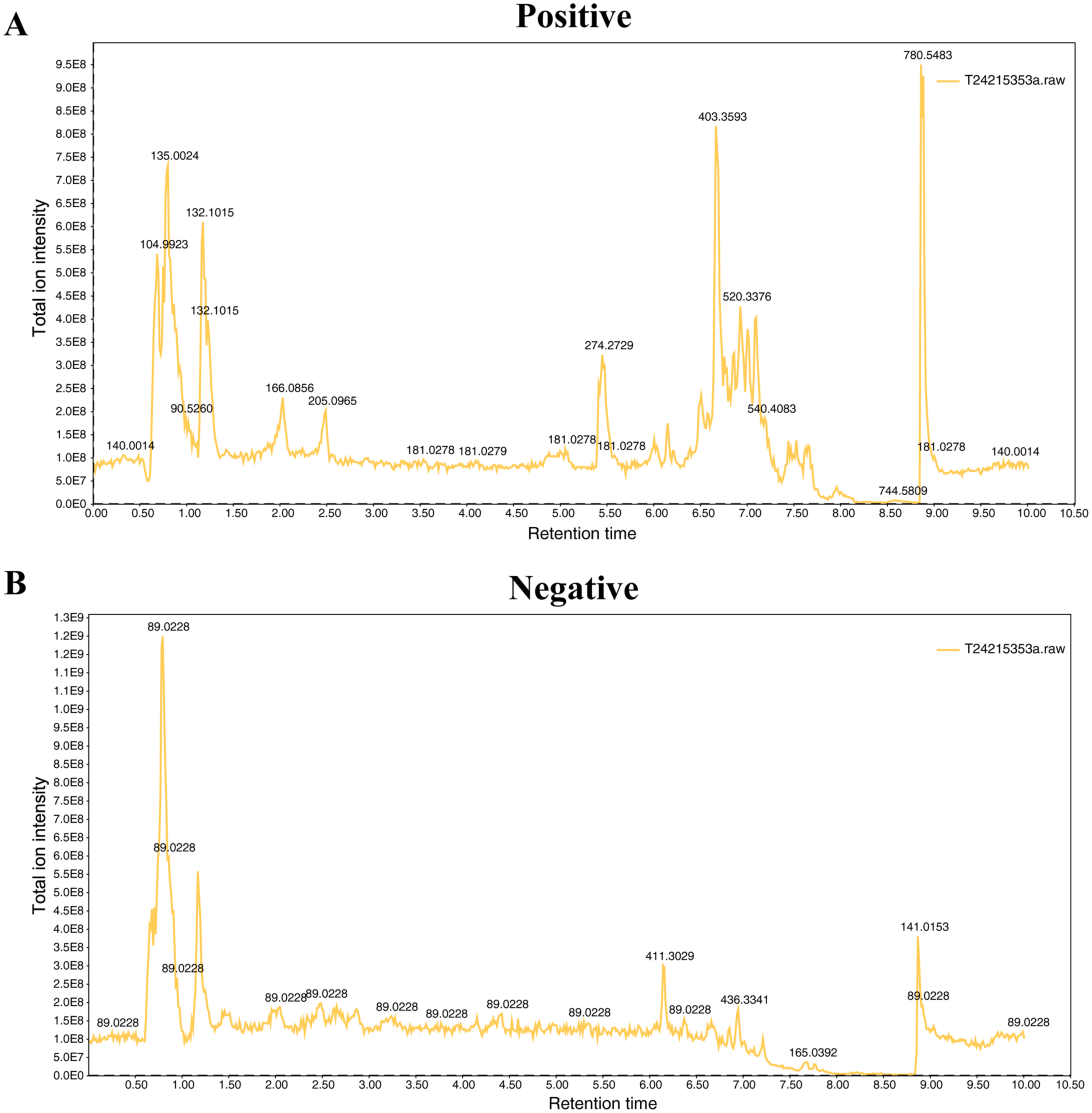
The representative total ion chromatograms of serum in positive **(A)** and negative **(B)** modes.

The representative total ion chromatograms (TICs) of serum in both positive and negative modes are displayed in Figure 1. As depicted in Figure 1, the well-defined and uniformly distributed peaks in the chromatogram demonstrate the exceptional separation capability of the T3 chromatographic column, confirming the validity of our experiment. To assess the reproducibility and stability of the experiment, quality control (QC) samples were randomly combined with the sequencing samples. For metabolomics, a QC sample is a pooled sample created by equally aliquoting and thoroughly mixing the tested samples. The overlap of all the QC chromatograms revealed no deviation in RT or peak height, as illustrated in Supplementary Figure 1. The complete concordance of the five QC samples provides evidence of the robustness and reliability of the entire experimental procedure and analysis process (Supplementary Figure 2).

### Global metabolomic changes with *Streptococcus pneumoniae* infection

**Figure 2:**
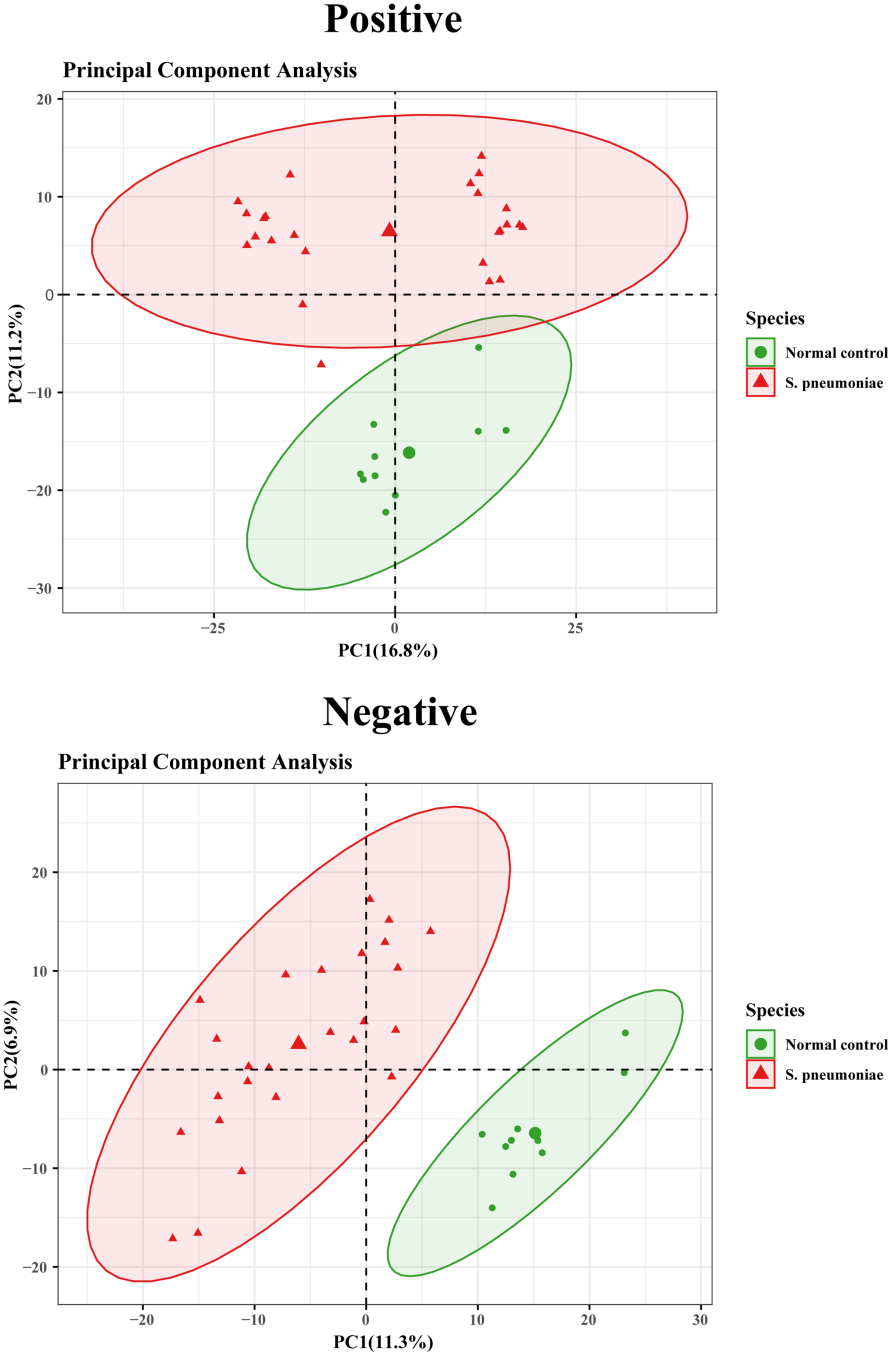
Score plots in positive mode and (right) negative mode of *S. pneumoniae* and the normal control samples (positive, principal component PC1 = 16.8%, PC2 = 11.2%; negative, PC1 = 11.3%, PC2 = 6.9%).

**Figure 3:**
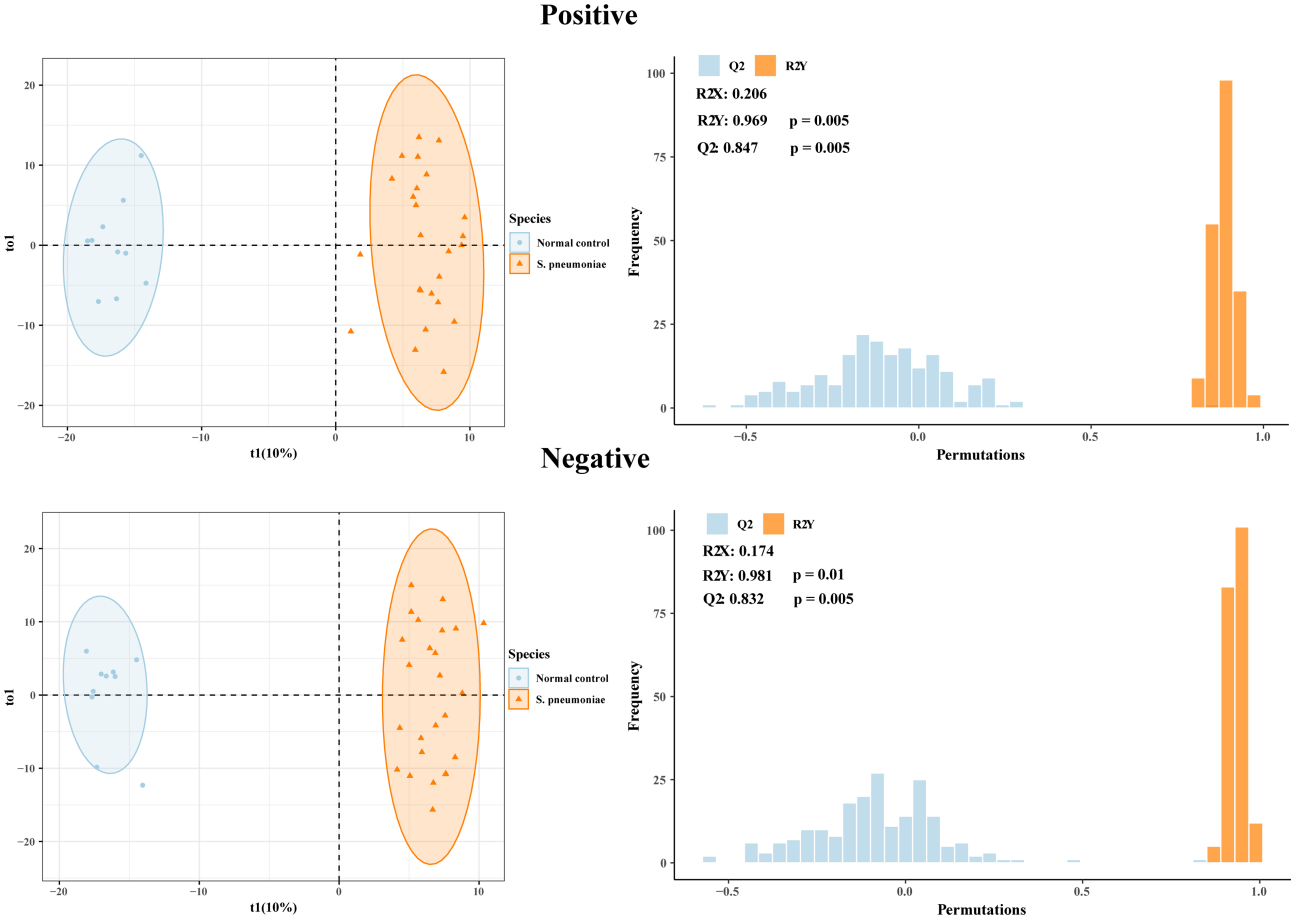
Orthogonal partial least squares discriminant analysis (OPLS-DA) score plots and permutation tests of the *S. pneumoniae* and normal control groups.

The PCA model strengthened the feasibility of the modeling process, with the corresponding parameters being PC1 = 16.8% and PC2 = 11.2% for the positive mode and PC1 = 11.3% and PC2 = 6.95% for the negative mode (Figure 2). These values indicate a satisfactory interpretative capacity of the model. The PCA results revealed a distinct separation in sample distribution between the *S. pneumoniae* group and the normal control group. To further identify differentially abundant metabolites contributing to this grouping, an OPLS model was employed in which the filtering criteria were VIP > 1 and *p* < 0.05. The OPLS analysis facilitated a more distinct grouping of the models. To ensure model reliability and reduce the risk of overfitting, external validation was conducted via a permutation test. The quality of the OPLS-DA model was evaluated by R2Y and Q2 parameters, which indicated the fitness and prediction of the model, respectively. Model performance was considered exemplary, as indicated by the R2Y and Q2 parameters, which were 0.969 and 0.847 in positive mode and 0.981 and 0.832 in negative mode, respectively, as shown in Figure 3.

### Identification of significantly altered metabolites and pathways

A total of 1756 metabolites were identified through interactive authority verification using a standard database in both positive and negative modes. When the filtering criteria were applied, 418 metabolites were identified, with 262 exhibiting an increasing trend and 156 showing a decreasing trend. Detailed information on the differentially expressed metabolites was presented in Supplementary Table 2.

**Figure 4:**
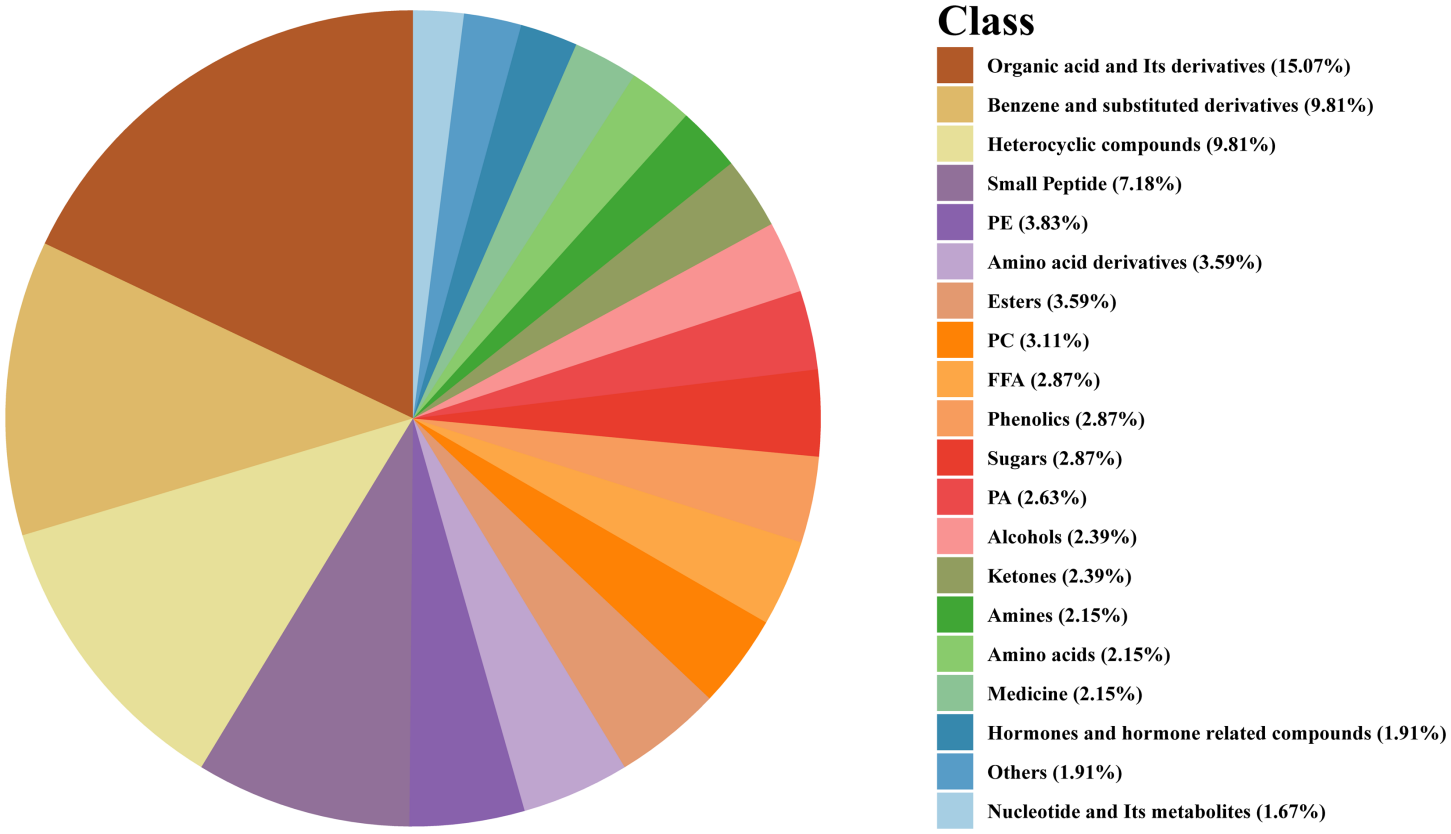
The classification of potential biomarkers.

**Figure 5:**
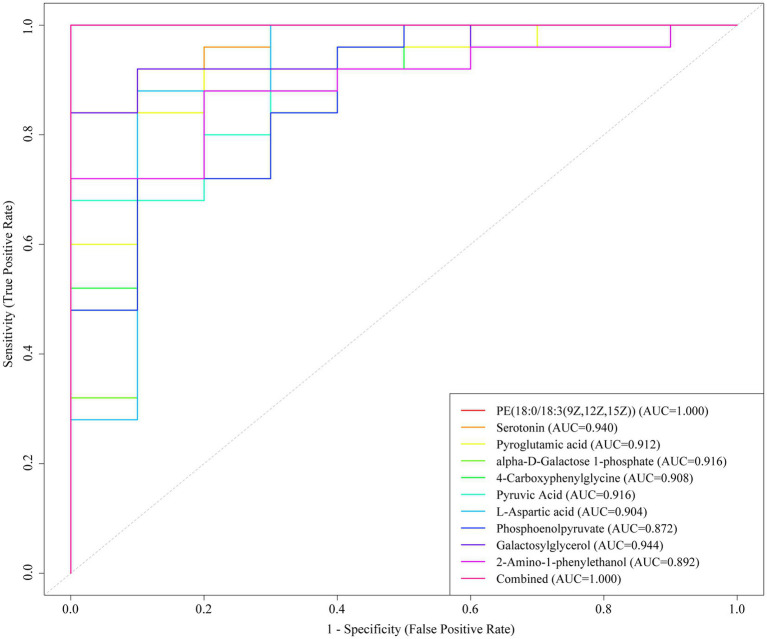
The area under the curve (AUC) of the analysis of the top 10 potential biomarkers.

The identified metabolites encompassed a diverse array of classes, including amino acids (AAs), fatty acids (FAs), phosphatidylcholine (PC), carbohydrates, acylcarnitines, and vitamins, as illustrated in Figure 4. In this study, the differentially abundant metabolites included certain drug metabolites and their metabolic products, which was attributable to the administration of pharmacological treatments to patients to combat the pathogenic infection following the onset of symptoms. Consequently, drugs were not the primary focus of our investigation; instead, we focused on the organism’s immune response to pathogen infection. The differential metabolites exhibited significant alterations between the *S. pneumoniae* group and the normal control group, suggesting their potential as diagnostic biomarkers and for elucidating disease pathogenesis. The diagnostic capability of the model with respect to the 10 potential biomarkers was illustrated in Figure 5. Representative differentially expressed metabolites were listed in Table 1.

**Table 1:** Representative differentially expressed metabolites.

Compounds	VIP	*P* value	Log2(FC)	Trend
PE (18:0/18:3(9Z,12Z,15Z))	2.83	0.00	5.55	↑
Serotonin	2.44	0.00	1.01	↑
Pyroglutamic acid	2.22	0.00	1.34	↑
alpha-D-Galactose 1-phosphate	2.21	0.00	1.14	↑
4-Carboxyphenylglycine	2.19	0.00	1.77	↑
Pyruvic acid	2.18	0.00	1.28	↑
L-Aspartic acid	2.17	0.00	0.99	↑
Phosphoenolpyruvate	2.11	0.00	1.17	↑
Galactosylglycerol	2.04	0.00	1.44	↑
2-Amino-1-phenylethanol	2.02	0.00	0.57	↑

**Figure 6:**
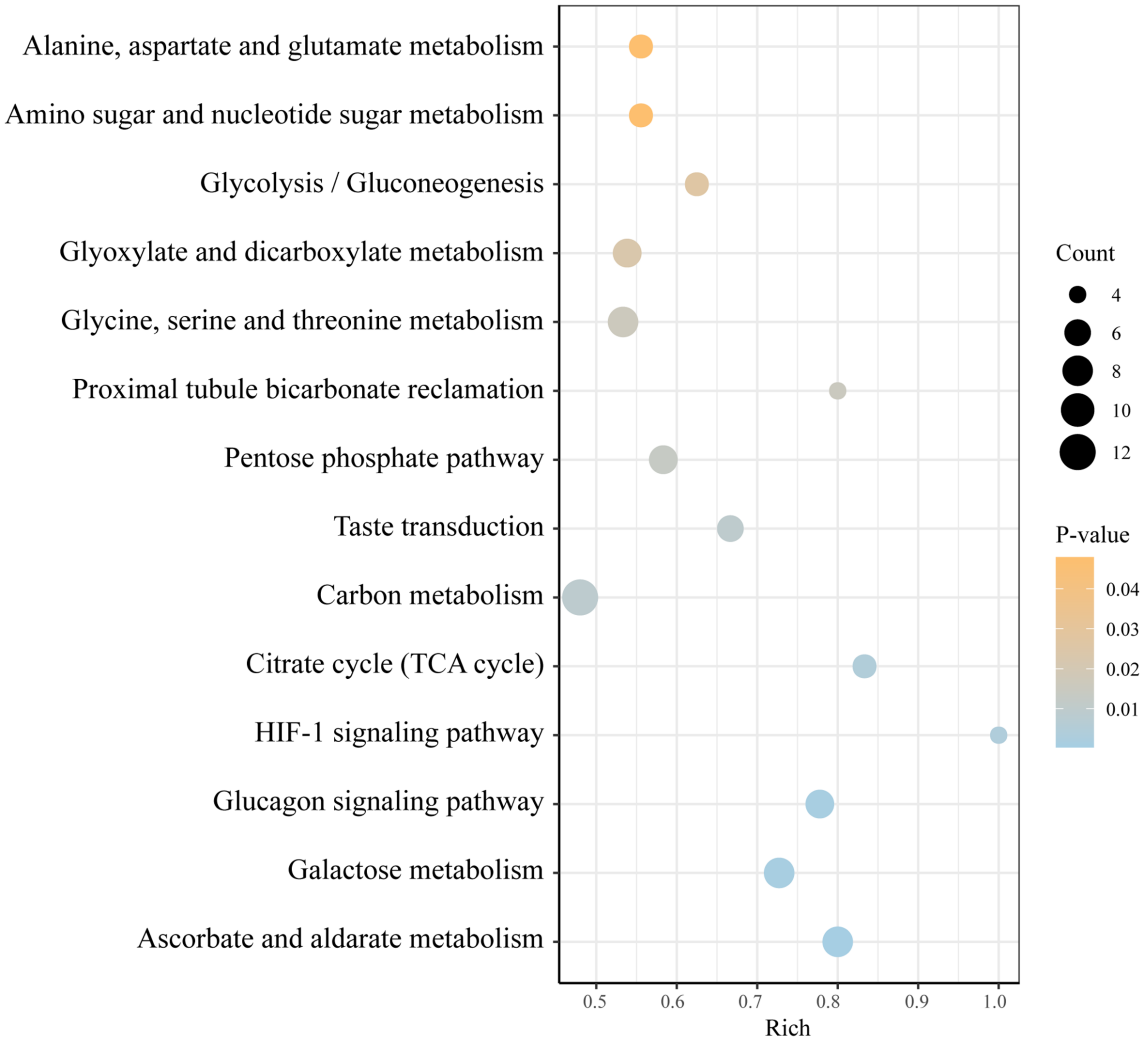
Kyoto Encyclopedia of Genes and Genomes (KEGG) enrichment pathways associated with the potential biomarkers detected in the *S. pneumoniae* and normal control samples.

**Figure 7:**
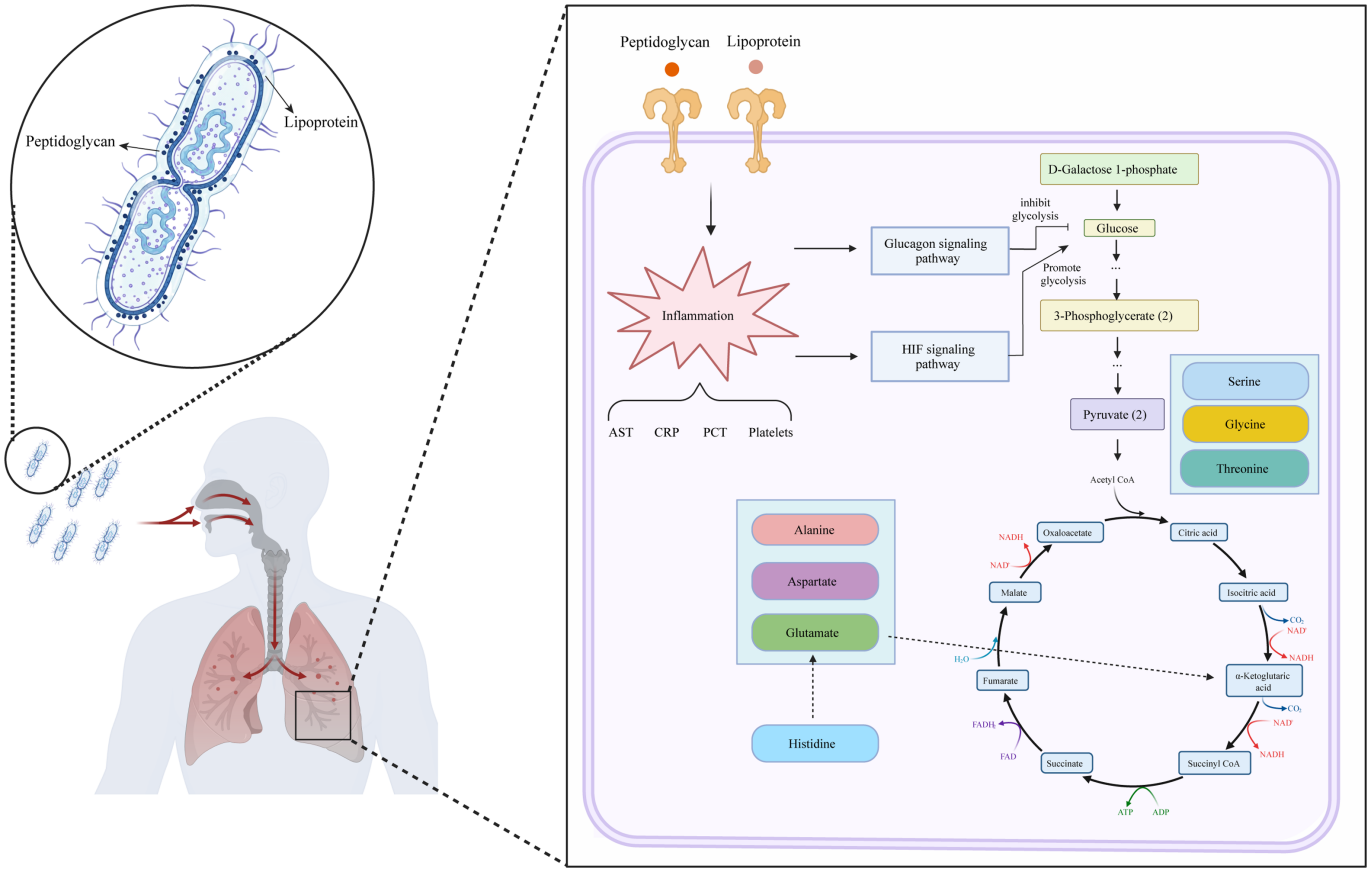
Metabolic profile map of *S. pneumoniae* infection.

The integration of potential metabolites was conducted using KEGG pathway enrichment analysis within the R studio environment. The analysis revealed several key metabolic pathways that were predominantly disordered, including the citrate cycle, the pentose phosphate pathway, glycolysis/gluconeogenesis, glycine, serine and threonine metabolism, alanine, aspartate and glutamate metabolism, histidine metabolism, and thiamine metabolism. Metabolic network analysis further revealed that the most significant disruptions were associated with glucose metabolism and AA metabolism pathways, both of which are intricately linked to energy metabolism and AA metabolism, playing crucial roles in the complex process of pathogen infection. A detailed depiction of these metabolic pathways is provided in Figure 6. The overview diagram of infection and the host main immune response is shown in Figure 7.

## Discussion

*Streptococcus pneumoniae* is a significant opportunistic pathogen in humans and is recognized as the primary cause of bacterial pneumonia worldwide, contributing substantially to morbidity and mortality among children through illnesses such as pneumonia, sepsis, and meningitis (Yang et al., 2024; Brizuela et al., 2024). This bacterium typically colonizes the human nasopharynx and upper airways but has the capacity to cause severe disease upon invasion of the lower respiratory tract (Prevotat et al., 2018), leading to symptoms such as fever, cough, and dyspnoea. In severe cases, *S. pneumoniae* infection can result in complications such as pneumonia, otitis media, and bacteraemia, thereby significantly impacting patients’ quality of life (Vaughn et al., 2024). The current standard treatment for infections caused by *S. pneumoniae* involves the administration of antibiotics, such as penicillin and related drugs. However, the increasing prevalence of antibiotic resistance presents new challenges in the management of these infections (Dai et al., 2023). Consequently, the prevention and treatment of *S. pneumoniae* infections remain critically important. An in-depth analysis of the complex mechanisms underlying *S. pneumoniae* infection, driven by factors such as bacterial adhesion and colonization, the role of the capsule, the inflammatory response, and host-related factors, is required to determine potential therapeutic targets. In this study, metabolomics was utilized to elucidate the comprehensive metabolic alterations that occur in patients with *S. pneumoniae* infection, and metabolic pathways modified during infection were identified. A cohort comprising 20 individuals infected with *S. pneumoniae* and 10 normal controls was analyzed, dynamic changes were revealed, and reliable differential information was extracted. Multivariate statistical analysis revealed a clear distinction between the *S. pneumoniae* group and the normal control group. However, the overall explanatory power of the model is relatively low. This may be attributed to the small sample size, which fails to adequately reflect the true differences between the *S. pneumoniae* group and the normal control group.

Metabolites exhibiting upwards and downwards trends in both positive and negative ion modes played crucial roles in the differentiation of group samples. Our investigation revealed significant alterations in approximately 400 serum metabolites associated with human metabolism. The altered metabolites may function as diagnostic biomarkers for the disease and offer insights into its pathophysiological progression. While these proof-of-concept biomarkers demonstrate promising diagnostic potential, their identification was based on a limited sample size. Therefore, additional validation studies with larger patient cohorts are warranted to confirm their clinical utility. Metabolomic profiling revealed significant alterations in carbon source-energy metabolism and AAs following *S. pneumoniae* infection. These pathway-derived metabolites may reflect host-pathogen interactions during immune activation. Ten candidate biomarkers exhibiting an area under the receiver operating characteristic curve (AUROC) > 0.85 were identified. Although these biomarkers lack specificity for *S. pneumoniae* infection individually, their diagnostic and prognostic utility may be enhanced when integrated with serological testing. Pneumococcal infection primarily activates the host innate immune system through pattern recognition receptors such as Toll-like receptors (TLRs). TLRs expressed in the respiratory tract recognize conserved components on the surface of *S. pneumoniae*, such as peptidoglycans and lipoproteins. This recognition initiates intracellular signaling cascades, leading to the production of proinflammatory cytokines and the subsequent recruitment of immune cells, which form the first line of defense against infection (Werren et al., 2023; Lane et al., 2022).

Although we did not measure key substances involved in the immune response in this study, we did focus on acute-phase reactants stimulated by cytokines, such as CRP and PCT, during this process (Yadav et al., 2024). Notably, both indicators were significantly elevated in the infected group, indicating an increase in the immune response. Notably, the absence of significant changes in peripheral WBC counts may be attributed primarily to the severity of the infection and the individual’s immune status. AST levels showed an upward trend compared with those in the normal control group, potentially due to mitochondrial damage in hepatocytes. In contrast, ALT levels remained stable, possibly because the primary site of damage is not the cytoplasm or because the extent of damage is insufficient to cause a significant change in ALT levels.

*Streptococcus pneumoniae*, a significant pathogenic bacterium, can proliferate in the lungs or other parts of the human body upon entry, initiating inflammatory responses and tissue damage. During the infection process, *S. pneumoniae* exploits host nutrients for growth and reproduction, thereby exacerbating infection symptoms. The fundamental prerequisite for *S. pneumoniae* infection is energy, which facilitates the uptake and utilization of glucose by the bacterium to support its proliferation. During pathogen infection, the host organism mounts a defense and generates an immune response to counteract the infection, resulting in an elevated energy demand. Consistent with Cheng’s findings, this immune activation is accompanied by a metabolic shift from oxidative phosphorylation to aerobic glycolysis, which constitutes a critical component of the initial host defense mechanisms (Cheng et al., 2016). To meet this increased energy requirement, the body may initiate gluconeogenesis to synthesize additional glucose as an energy source (Dhaliwal et al., 2024). Our research demonstrated that key metabolites involved in the glucose metabolic pathway, including glucose, pyruvate, and phosphoenolpyruvate, exhibit varying degrees of increase or decrease. This observation aligns with findings that the transition to glycolysis is associated with the sufficient production of adenosine triphosphate (ATP) and biosynthetic intermediates, enabling the execution of distinct functional roles such as phagocytosis and antimicrobial responses (Figueirêdo Leite et al., 2023). The tricarboxylic acid (TCA) cycle, which is a metabolic pathway downstream of glucose metabolism, plays a pivotal role in organisms. The TCA cycle serves as a pivotal nexus interlinking the metabolism of carbohydrates, lipids, and AAs while also exhibiting a significant association with the metabolic alterations that transpire in the host during pathogen infection (Chen et al., 2023). In addition to its fundamental role in energy metabolism, the TCA cycle uses its intermediate metabolites and derivatives as signaling molecules that modulate immune cell functions (Yang et al., 2024).

The hypoxia-inducible factor-1 (HIF-1) signaling pathway involves critical transcription factors that respond to hypoxic environmental changes and play a vital role in the host immune response. HIF-1 is instrumental in the immune response to external infectious agents by facilitating metabolic reprogramming and the inflammatory response of immune cells (Shi et al., 2023). An active HIF-1complex specifically binds to hypoxic response elements on DNA, initiating transcription of genes crucial for biological processes like angiogenesis, energy metabolism, and cell survival (Chen et al., 2022). Research indicates that during the initial stages of *Mycobacterium tuberculosis* infection, macrophages undergo significant metabolic alterations, characterized by increased glycolytic flux and a decrease in mitochondrial oxidative phosphorylation, alongside an upregulation of HIF-1α expression. This metabolic shift parallels the “Warburg effect” observed in the tumour microenvironment, wherein the activation of host innate and adaptive immune cells is accompanied by a transition from oxidative phosphorylation to glycolysis as the primary bioenergetic pathway (Kim et al., 2020). HIF-1α modulates the energy metabolism of macrophages to adapt to hypoxic environments and inflammatory conditions by promoting glycolytic processes and limiting mitochondrial aerobic respiration.

HIF-1 is pivotal in regulating the energy metabolism of immune cells, exerting its effects through the modulation of various molecular pathways (Wang et al., 2024; Xiang et al., 2025). HIF-1 influences the type of energy metabolism in immune cells by activating specific downstream signaling pathways, including the AMP-activated protein kinase (AMPK) pathway. AMPK, a crucial regulator of cellular energy metabolism, is activated under conditions of energy deficiency, thereby promoting energy production and reducing energy expenditure. Research indicates that the AMPK/HIF-1 pathway exerts anti-inflammatory effects on the regulation of immune cell energy metabolism (Yu et al., 2020). These findings suggest that the AMPK/HIF-1 pathway not only governs the energy metabolism of immune cells but also potentially influences the regulation of inflammatory diseases. In this study, metabolites, such as pyruvate, adenosine diphosphate (ADP), and adenosine monophosphate (AMP), were enriched in the AMPK pathway, further corroborating the link between AMPK and energy metabolism.

Our serum metabolic analysis further demonstrated that the majority of metabolites linked to AA metabolism, including glycine, serine, and threonine metabolism; alanine, aspartate, and glutamate metabolism; and histidine metabolism, were affected. Dysregulation of these AAs suggests increased protein catabolism and is indicative of *S. pneumoniae* pathology. Pathway analysis revealed an enrichment of glycine, serine, and threonine metabolism, which suggests increased glucose uptake and glycolysis (Codo et al., 2020). This finding aligns with the primary focus of the present study on glucose metabolism. AA metabolism is crucial for immune function; beyond serving as mere nutrients for protein synthesis, amino acids are instrumental in directing cellular proliferation and effector functions (Shi et al., 2019). Histidine levels have been shown to correlate with increased immune function, potentially reducing the risk of pathogen infection (Wu et al., 2021). The metabolism of alanine, aspartate, and glutamate has been associated with the regulation of inflammatory and autoimmune responses (Li et al., 2007), which is consistent with the findings of Li et al., indicating the activation of shared immune systems against pathogen infection and invasion (Chen et al., 2024). Aspartate plays a critical role in the metabolism and function of leukocytes and serves as a substrate for the synthesis of purine and pyrimidine nucleotides, which are essential for lymphocyte proliferation. Additionally, low alanine levels have been implicated in the development of immune system diseases (Murgia et al., 2018). In this study, we did not observe differences in ALT levels between the infected and normal control groups, which may be attributed to the fact that metabolomic analysis primarily captures metabolite variations at specific time points.

## Conclusion

The findings of the present study regarding metabolic alterations have the potential to improve our currently limited understanding of the immune response to *S. pneumoniae*. The field of metabolomics offers a robust approach for elucidating the underlying mechanisms of *S. pneumoniae* infection and identifying therapeutic targets. Pathway enrichment analyses revealed a strong association between *S. pneumoniae* infection and glycolytic metabolism, highlighting potential areas for disease management in the future. The application of metabolomic techniques holds promise for providing a comprehensive metabolic characterization of bacterial infections, which could facilitate the development of improved diagnostic and therapeutic strategies. Future studies integrating multi-omics datasets and larger clinical cohorts could validate the identified biomarkers for diagnostic translation. Nonetheless, this study is subject to several limitations, including a small sample size, particularly concerning clinical specimens, which may affect the reproducibility of the metabolic biomarker results. Additionally, the complex and multifaceted nature of the immune response poses challenges in pinpointing optimal biomarkers for the immune response in cellular and tissue contexts. We chose a variety of annotated metabolic substances, uncovering specific constraints in the regulation of the immune response within the system.

## Data Availability

Data is available within the article or its Supplementary materials. All raw data were deposited in Mass Spectrometry Interactive Virtual Environment under accession number MSV000099197 for metabolomics data.

## Footnotes

1
https://hmdb.ca
