# Supplementary figures and images for: Metabolomic analysis of Streptococcus pneumoniae: uncovering key metabolic pathways
Source: Front Microbiol. 2025 Dec 4;16:1707940. doi: 10.3389/fmicb.2025.1707940 (PMC12711820; doi:10.3389/fmicb.2025.1707940)

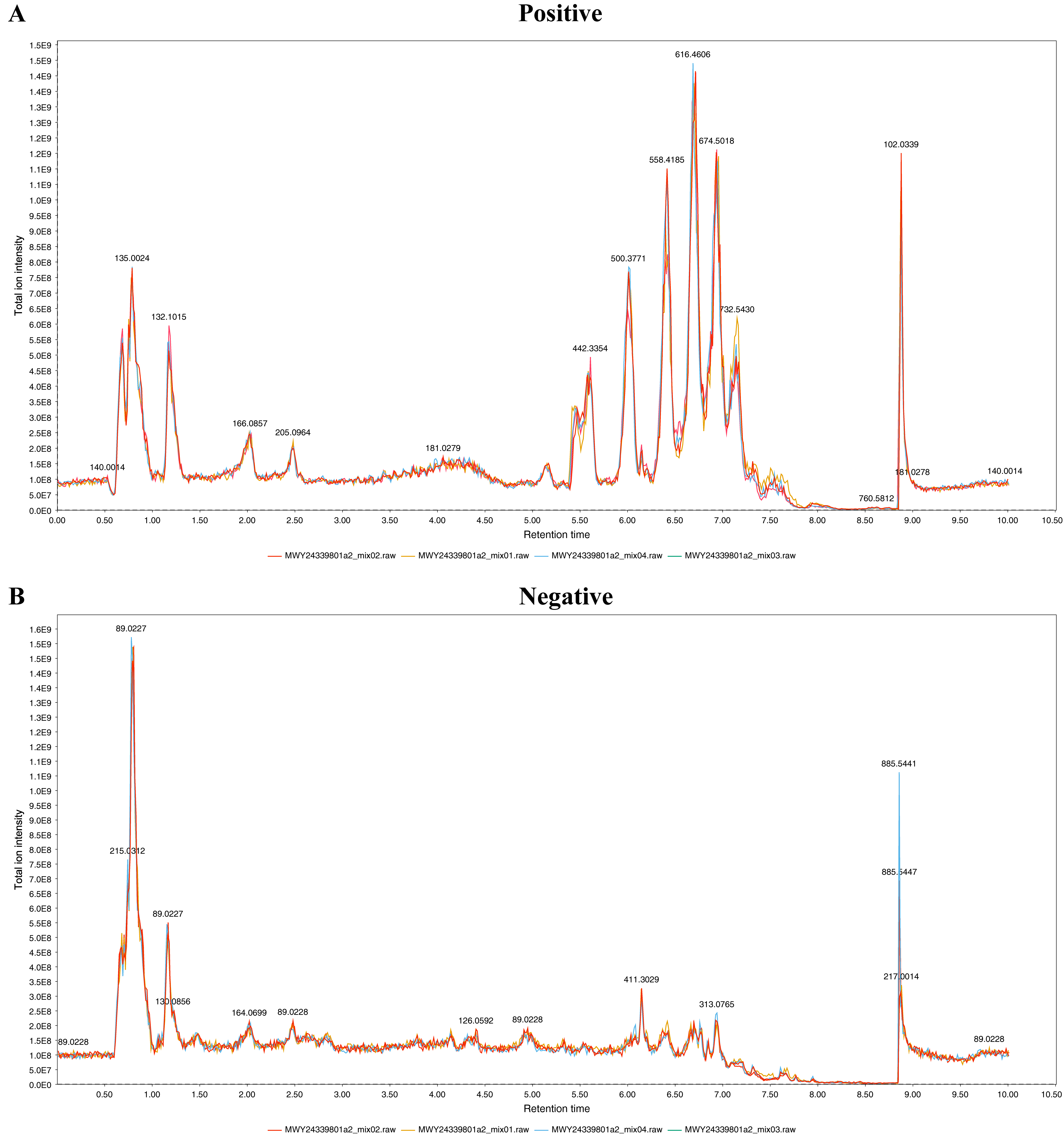

Supplement: Supplementary file 1 [file Image_1.tif]

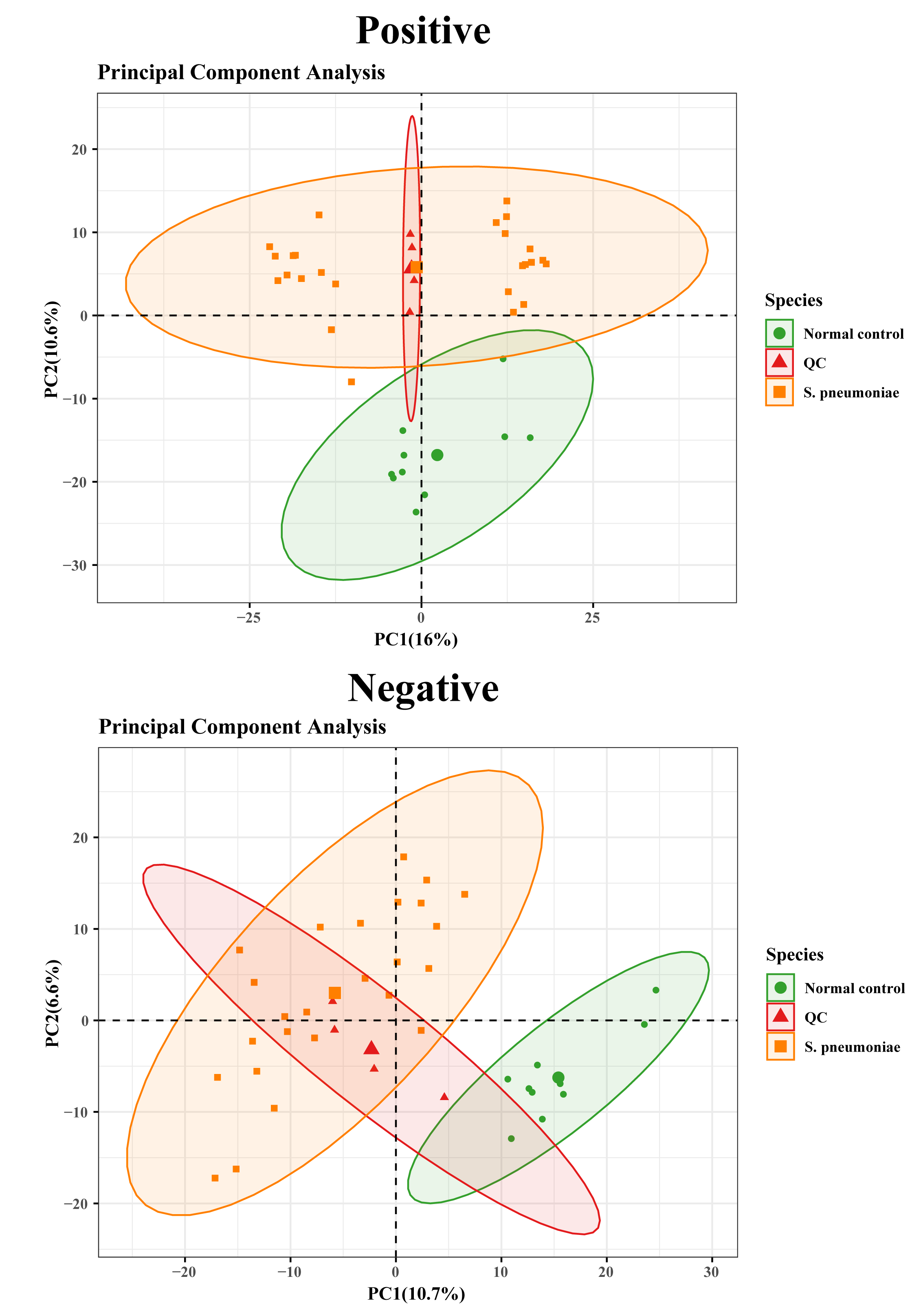

Supplement: Supplementary file 2 [file Image_2.tif]
