# Supplementary material for: Metabolomic analysis of Streptococcus pneumoniae: uncovering key metabolic pathways
Source: Front Microbiol. 2025 Dec 4;16:1707940. doi: 10.3389/fmicb.2025.1707940 (PMC12711820; doi:10.3389/fmicb.2025.1707940)
Supplement: Supplementary file 5 [file Table_3.docx]

***Supplementary Material***

**Metabolomic analysis of Streptococcus pneumoniae: Uncovering key metabolic pathways**

**HaiYan Jiang1, ChangLiang Zhao1, Lu Feng1, YunJun Gao1, ZhiHui Mi2,**

**HaiYing He1**

***Correspondence**

**HaiYing He, Email: he7004@126.com**

**Supplementary Table Captions**

**Supplementary Table 1. Detailed clinical information of the enrolled participants.**

**Supplementary Table 2. The differentially expressed metabolites of the *S. pneumoniae* group and the normal group.**

**Supplementary Figure 1. Overlap chromatograms of the quality control (QC) samples.**

**Supplementary Figure 2. Score plots in positive mode and negative mode classifying *S. pneumoniae*, normal control and quality control (QC) samples (positive, principal component [PC]1=16%, PC2=10.6%; negative, PC1=10.7%, PC2=6.6%).**
